# Immediate and Longitudinal Alterations of Functional Networks after Thalamotomy in Essential Tremor

**DOI:** 10.3389/fneur.2016.00184

**Published:** 2016-10-24

**Authors:** Changwon Jang, Hae-Jeong Park, Won Seok Chang, Chongwon Pae, Jin Woo Chang

**Affiliations:** ^1^BK21 PLUS Project for Medical Science, Severance Hospital, Yonsei University College of Medicine, Seoul, South Korea; ^2^Department of Nuclear Medicine, Severance Hospital, Yonsei University College of Medicine, Seoul, South Korea; ^3^Department of Radiology, Severance Hospital, Yonsei University College of Medicine, Seoul, South Korea; ^4^Department of Psychiatry, Severance Hospital, Yonsei University College of Medicine, Seoul, South Korea; ^5^Department of Cognitive Science, Yonsei University, Seoul, South Korea; ^6^Department of Neurosurgery, Yonsei University College of Medicine, Seoul, South Korea

**Keywords:** thalamotomy, essential tremor, brain network, diaschisis, connectivity

## Abstract

Thalamotomy at the ventralis intermedius nucleus has been an effective treatment method for essential tremor, but how the brain network changes immediately responding to this deliberate lesion and then reorganizes afterwards are not clear. Taking advantage of a non-cranium-opening MRI-guided focused ultrasound ablation technique, we investigated functional network changes due to a focal lesion. To classify the diverse time courses of those network changes with respect to symptom-related long-lasting treatment effects and symptom-unrelated transient effects, we applied graph-theoretic analyses to longitudinal resting-state functional magnetic resonance imaging data before and 1 day, 7 days, and 3 months after thalamotomy with essential tremor. We found reduced average connections among the motor-related areas, reduced connectivity between substantia nigra and external globus pallidum and reduced total connection in the thalamus after thalamotomy, which are all associated with clinical rating scales. The average connectivity among whole brain regions and inter-hemispheric network asymmetry show symptom-unrelated transient increases, indicating temporary reconfiguration of the whole brain network. In summary, thalamotomy regulates interactions over the motor network via symptom-related connectivity changes but accompanies transient, symptom-unrelated diaschisis in the global brain network. This study suggests the significance of longitudinal network analysis, combined with minimal-invasive treatment techniques, in understanding time-dependent diaschisis in the brain network due to a focal lesion.

## Introduction

The current prevailing view of the brain is that it is not simply a mixture of isolated regions but a highly organized system of interactions. Analysis of network topology in the brain (1) highlights the properties of the system, such as efficient information passing, adaptability, and resilience (2). From theoretical modeling and clinical studies, these system properties explain the greater vulnerability and detrimental topological reorganization following damage to the neuronal hubs (3–5). The network centric perspective is receiving increasing attention in understanding brain diseases and treatments, particularly in investigating how functional networks respond immediately to insults (or treatments) in the anatomical network and then reorganize afterwards.

From this network perspective, we investigated changes in the brain networks of patients with essential tremor following thalamotomy. Essential tremor, which manifests as involuntary trembling of the body or limbs, is the most common adult movement disorder with a population incidence of ~0.7%, rising to 4.6% in those above the age of 65 years (6). To treat essential tremor, surgical interventions, such as thalamotomy and deep brain stimulation, have been used (7–10). However, how a deliberate lesion to a focal target leads to immediate changes in the motor network and alleviates tremor remains poorly understood.

Researches on the immediate changes in the brain network following surgical lesioning have been hampered by several limitations inherent in invasive techniques, such as inevitable impairments to non-target regions and potential side effects due to cranium opening. The recently established magnetic resonance imaging-guided high-intensity focused ultrasound (MRgFUS) thermal ablation technique provides a unique chance to view abrupt changes in the brain network caused by a focal lesion. MRgFUS ablates a target region using focused sonification without cranium-opening under the guidance of real-time MRI temperature mapping for targeting and has shown its efficacy in thalamotomy for essential tremor (11–14).

To investigate the effects of thalamotomy on the brain network, we combined this MRgFUS technique and resting-state functional magnetic resonance imaging (rs-fMRI). rs-fMRI makes it possible to construct functional networks utilizing low-frequency clustered endogenous fluctuations of the blood oxygenation level-dependent (BOLD) signals at rest (15).

In this study, we investigated alterations of the motor area and the whole brain with respect to the network rather than regional activities at the target. Since a network is more than a sum of edges [functional connectivity (FC) between two regions], a change in an edge should be considered in the context of the network to which it belongs and when it is involved (16). Thus, we utilized graph theory methods to evaluate network changes more than focusing on a single edge.

We also investigated changes in the motor and global brain networks with respect to time course. Instant changes of brain networks following thalamotomy for essential tremor are typically as a result of mixed factors, such as symptom-related treatment effects and symptom-unrelated (or indirectly related) transient effects. Differentiation of the two main factors and specifying the main treatment effects of thalamotomy requires longitudinal follow-up. Accordingly, we conducted network analyses of rs-fMRI acquired prior to surgical treatment, as well as 1 day, 7 days, and 3 months after thalamotomy using MRgFUS. We expected the network features corresponding to short-term transient changes to recover while those associated with direct treatment effects would be maintained in line with the level of symptoms. We generally hypothesized that focal lesioning would affect global networks other than connectivity directly associated with the target lesion due to the network nature of the brain.

## Materials and Methods

### Subjects and MRgFUS Thalamotomy

Ten patients with essential tremor (mean age 65 years; one female) underwent left thalamotomy by thermally ablating the target in the ventralis intermedius nucleus (Vim) of the thalamus using MRgFUS. Thalamotomy using MRgFUS failed in two patients due to insufficient increase in the temperature at the target. Eight out of ten patients completed four sessions of rs-fMRI scanning: before treatment (op − 1d), 1 day after treatment (op + 1d), 7 days after treatment (op + 7d), and 3 months after treatment (op + 3m). The degree of tremor was evaluated using the Clinical Rating Scale for Tremor Part A (CRST A) (17). The right hand CRST A action score represents the degree of tremor for patients moving their hands, while CRST A posture score represents the tremor score without moving. All patients received the standard clinical and imaging workup as part of the study’s baseline requirements. In the current study, we continued the use of same medications for each patient if a patient was on any prescribed medications before treatment. This was to control for the effects of medications in our evaluation of treatment effects. All patients provided written informed consent before procedures and this study received full ethics approval from the Korean Food and Drug Administration (KFDA) and Institutional Review Board of Yonsei University Severance Hospital and the Declaration of Helsinki (World Medical Association, 1964, 2008).

**Figure 1:**
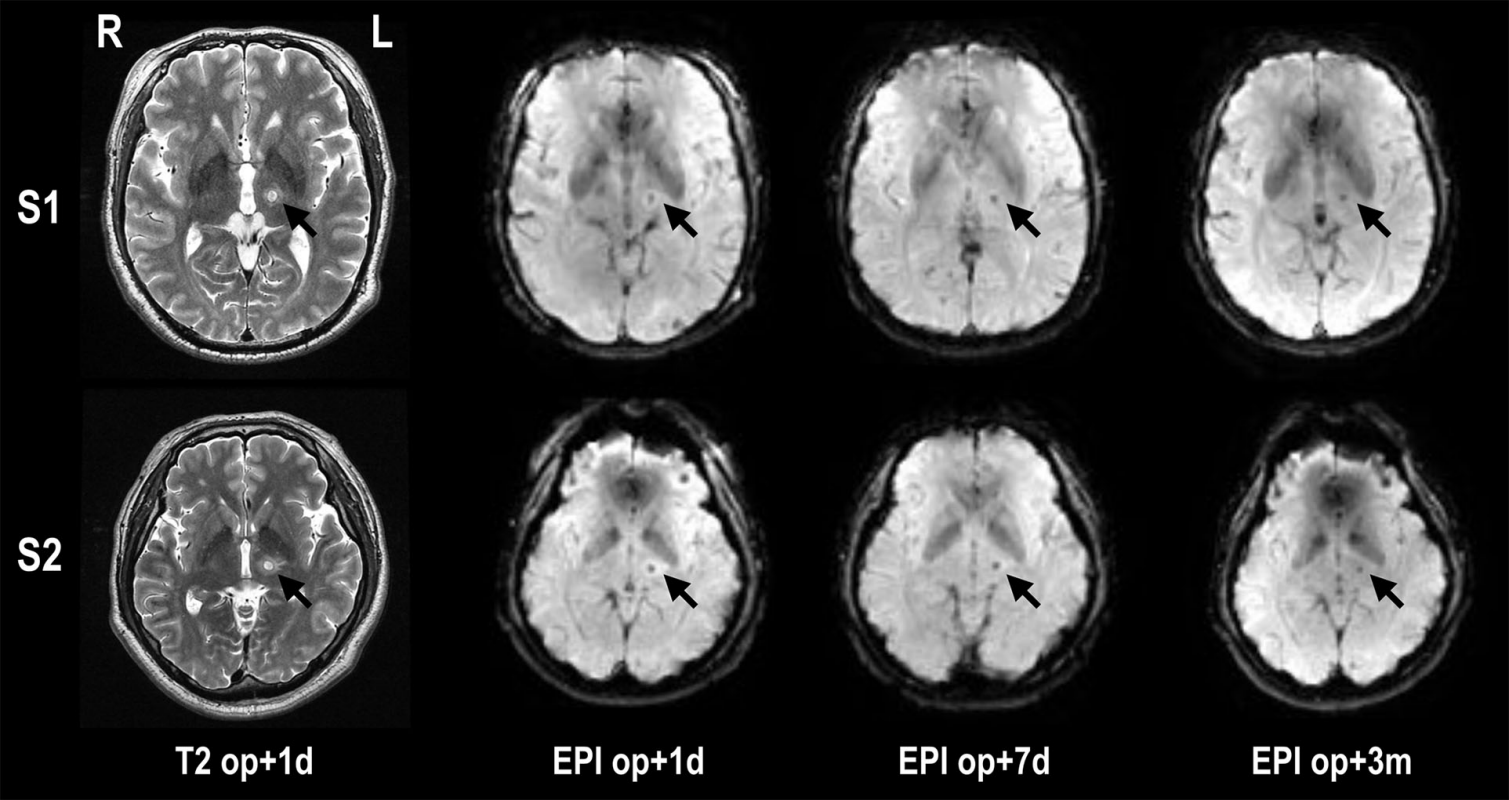
**Lesion locations in T2-weighted images and fMRI echo planar images after MRgFUS thalamotomy in two patients**. The target lesion is the left ventralis intermedius nucleus (Vim) of the thalamus (arrow). The temperature of the target region was increased until reaching a minimum 55°C, creating a lesion for each patient. op + 1d, op + 7d, and op + 3m indicate 1 day, 7 days, and 3 months after treatment, respectively.

All MRgFUS procedures were performed using ExAblate 4000 (InSightec, Tirat Carmel, Israel), which is integrated with a 3-T MRI (GE, Milwaukee, WI, USA) and destroys tissues by focusing a high-energy beam on the Vim of the thalamus and raising its temperature upto the range of 55–60°C. Figure 1 shows exemplar displays of MRgFUS ablation in two patients. Details of the procedure can be found in Chang et al. (13).

### Data Acquisition and Processing

Resting-state functional magnetic resonance imaging data were acquired axially using T2* weighted single shot echo planar imaging (EPI) sequences using a 3.0-T GE 750 MRI scanner (GE, Milwaukee, WI, USA) using a 16 channel head neck spine (HNS) array coil with the following parameters: voxel size, 1.88 mm × 1.88 mm × 4.5 mm; slice number, 30 (interleaved); matrix, 128 × 128; slice thickness, 4 mm; repetition time (TR), 2000 ms; echo time (TE), 30 ms; flip angle (FA) = 90°; and field of view, 240 mm × 240 mm. Each 330-s scan produced 165 fMRI images. Foam pads were used to reduce head motion during EPI data acquisition. Subjects were instructed to keep their eyes closed, without sleeping or specific thinking. After scanning, subjects were asked to report their sleepiness and general condition.

A high-resolution structural data set was also obtained from each subject using a 3D T1-BRAVO sequence with the following parameters: voxel size 0.43 mm × 0.43 mm × 1 mm; matrix size 516 × 516; FOV 220 mm × 220 mm; TR 8.59 ms; TE 3.32 ms; FA 12°.

All image processing and network analysis were based on the in-house software for multimodal network analysis.^1^ We used the Harvard-Oxford atlas^2^ to define brain nodes for network analysis.

Spatial preprocessing of fMRI data was conducted using statistical parametric mapping (SPM12,^3^ Wellcome Trust Centre for Neuroimaging, London, UK) (18). All EPI data were undergone standard preprocessing steps, including correction of acquisition time delays between different slices, correction for head motion by realigning all consecutive volumes to the first image of the session, and non-linear coregistration of T1-weighted image to the first EPI data. The non-linear coregistration algorithm was used to minimize image distortions in the EPI data by maximizing normalized mutual information between EPI and T1-weighted images using a modified version of Accelerated Image Registration with Compute Unified Device Architecture.^4^

Co-registered T1-image was used to spatially normalize functional EPI into the Montreal Neurological Institute (MNI) template space using non-linear transformation in SPM12. All registration steps were visually confirmed and semi-automatically adjusted in the case of mis-registration.

Functional magnetic resonance imaging time series for 110 cerebral brain regions of the Harvard-Oxford atlas were extracted from the normalized fMRI data in the MNI template space. Time series of eigenvalues corresponding to the first eigenvector, i.e., the mode, of time series for multiple voxels in each region (extracted using principal component analysis) was used as a representative activity of the region rather than the average of the voxels (19, 20). After discarding first five scans to address stability issues, we preprocessed fMRI time series by regressing out effects of six rigid motions and their derivatives and three principal components of the white matter and the cerebrospinal fluid masks (21). fMRI time series were again detrended by linear and quadratic repressors and high frequency filtered by regressing out cosine and sin waveforms up to 0.009 Hz (22). We did not filter higher frequency signals since higher frequency components (>0.1 Hz) of BOLD signals are known to carry meaningful information (23, 24).

### Construction of Functional Networks for the Whole Cerebral Brain

To define functional network, we used Pearson cross-correlation coefficient between time series of two nodes, a widely used measure for FC (15, 25, 26). This functional connectivity between two nodes is called an edge in the network. After calculating functional connectivity matrix for all 110 nodes, we converted cross-correlation coefficients to *Z*-values using Fisher’s *z*-transformation. Functional networks were created after thresholding (positive) connectivity matrix with a criterion of false discovery rate (FDR) (27) *q* < 0.05. This procedure for functional network construction was applied to all the data acquired at four sessions.

In the whole brain network analysis, we also used partial correlation as an index for direct functional connectivity (pFC). The functional connectivity using Pearson correlation reflects both direct interactions and indirect polysynaptic interactions or modulation effects of a third node between the two nodes. Instead, partial correlation is more efficient in revealing direct associations between the brain areas (28, 29). We calculated the partial-correlation matrix of the mode time series among the 110 regions using the graphical least absolute shrinkage and selection operator (gLASSO) technique (30). As a gLASSO method, we used sparse inverse covariance estimation (31). The regularization parameter λ was determined using Stability Approach to Regularization Selection (32). Since edges generally shrink after gLASSO, we adjusted the edges to reflect connectivity strength properly (33) using a sample covariance matrix and a sparse structure of original precision matrix (34). Functional networks were constructed by thresholding pFC > 10^−5^, since gLASSO shrinks unrelated edges to 0.

**Figure 2:**
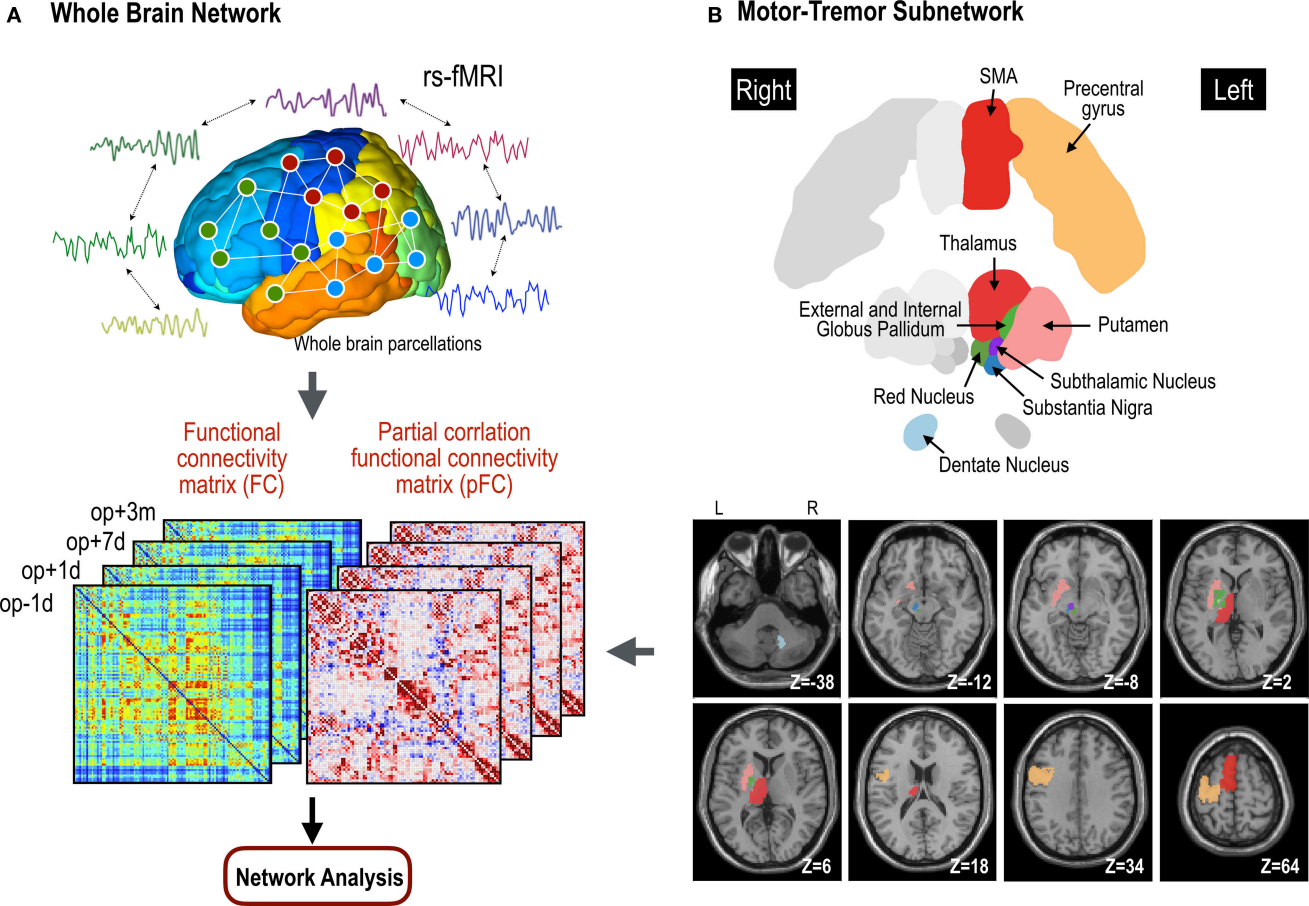
**Procedures for network analyses in the whole brain network (A) and motor subnetwork (B)**. The whole brain network is composed of 110 nodes in the cerebral cortex. Cross-correlation and partial cross-correlation of the time series across all nodes compose functional connectivity (FC) and partial-correlation functional connectivity (pFC) matrices at four time points: op − 1d, op + 1d, op + 7d, and op + 3m (1 day before operation, 1 day after operation, 7 days after operation, and 3 months after operation, respectively). The same procedure for functional network analysis was done within the motor subnetwork **(B)**, except for structure–function coupling. The motor subnetwork includes the precentral gyrus, supplementary motor areas (SMA), putamen, thalamus, external globus pallidum, internal globus pallidum, subthalamic nucleus, substantia nigra, and red nucleus in the left hemisphere, and right dentate nucleus in the cerebellum.

We used both FC and pFC in the analysis of whole brain functional networks since they designate slightly different properties of functional networks as explained above. All the process is summarized in Figure 2.

### Functional Motor-Tremor Networks

We constructed a functional motor-tremor subnetwork for each individual in each session. The motor network included 10 brain regions: the left precentral gyrus, left supplementary motor area, left thalamus, left putamen, left internal globus pallidum, left external globus pallidum, left subthalamic nucleus, left substantia nigra, left red nucleus, and right dentate nucleus (Figure 2). The precentral gyrus, thalamus, and dentate nucleus are known to be involved in the tremor circuit (35). We used regions for the red nucleus, substantia nucleus, sub-thalamic nucleus, and the internal/external globus pallidum as detailed in the ATAG atlas (36). We manually delineated the dentate nucleus of each patient according to the method detailed by Tellmann et al. (37). Using a time series for each region, functional networks for motor areas were constructed using the same method used for whole brain functional network construction.

### Graph-Theoretic Properties of Nodes, Edges, and Networks

Graph-theoretic measures for brain networks (2) were calculated using BCT toolbox (38) for functional networks of the whole brain and motor-related areas. In the application of graph theory for the human connectome, measures for network properties are initially developed on the structural network. However, the same measures are also meaningful on the functional network as an index for evaluating interactions over the structural network as is conventionally used in the social network analysis.

(1) *Nodes*: we calculated node degree and path-length at each node. The degree of a node is the number of connections that link the node and the rest of the network as a most fundamental network measure (2). The node strength is the sum of all connections with the node. The path-length of a node is the average of the minimum number of edges to connect different nodes (39). Except for node degree, node strength, and path-length were evaluated in the weighted networks of FC and pFC. We focused on changes in the functional connectivity particularly in the target lesion, i.e., the left thalamus.
(2) *Edges*: we also compared connectivity strengths at all edges defined by FC and pFC according to acquisition sessions.
(3) *Networks*: as global network properties, we calculated inter-hemispheric network similarity, average node degree, average node strength and global efficiency of functional networks before and after the thalamotomy. The inter-hemispheric network similarity was defined by a cross-correlation coefficient between edges (elements of the connectivity matrix) in the left and right brain subnetworks. The average node degree indicates the average number of existed interaction among all pairs of nodes in the network. The average node strength designates the sum of functional connectivity among all pairs of nodes in the network. The global efficiency is the average inverse shortest path-length across the network, indicating efficiency of information passing.

### Statistical Analysis of Network Properties

We compared all graph-theoretic properties (nodal properties, edge strength, network properties) by session using repeated-measures analysis of variance (ANOVA). In the *post hoc* analysis of ANOVA, we used Tukey’s HSD (honest significant different) test to adjust for issues regarding multiple testing problem. As a *post hoc*, we also used a linear mixed effect model to test whether there is a linear relationship between network properties and motor symptom scales, i.e., CRST A action and posture scores. The linear mixed effect model was defined as below:

y=Xβ+Zγ+ε

where *y* denotes a network property as an outcome variable, X is a vector of predictor variables (action and posture scores), β is the fixed-effect regression coefficient, Z is the design matrix for random effects, γ represents random effects, and t is the unknown vector of random errors. We used SPSS software (SPSS, Inc., Chicago, IL, USA) with patients as random effects and CRST A scores as fixed effects. To correct for multiple testing in the network analysis, we used a FDR of *q* < 0.05 to control the error rate in the analysis of nodes and edges.

## Results

### Demographic Data

**Figure 3:**
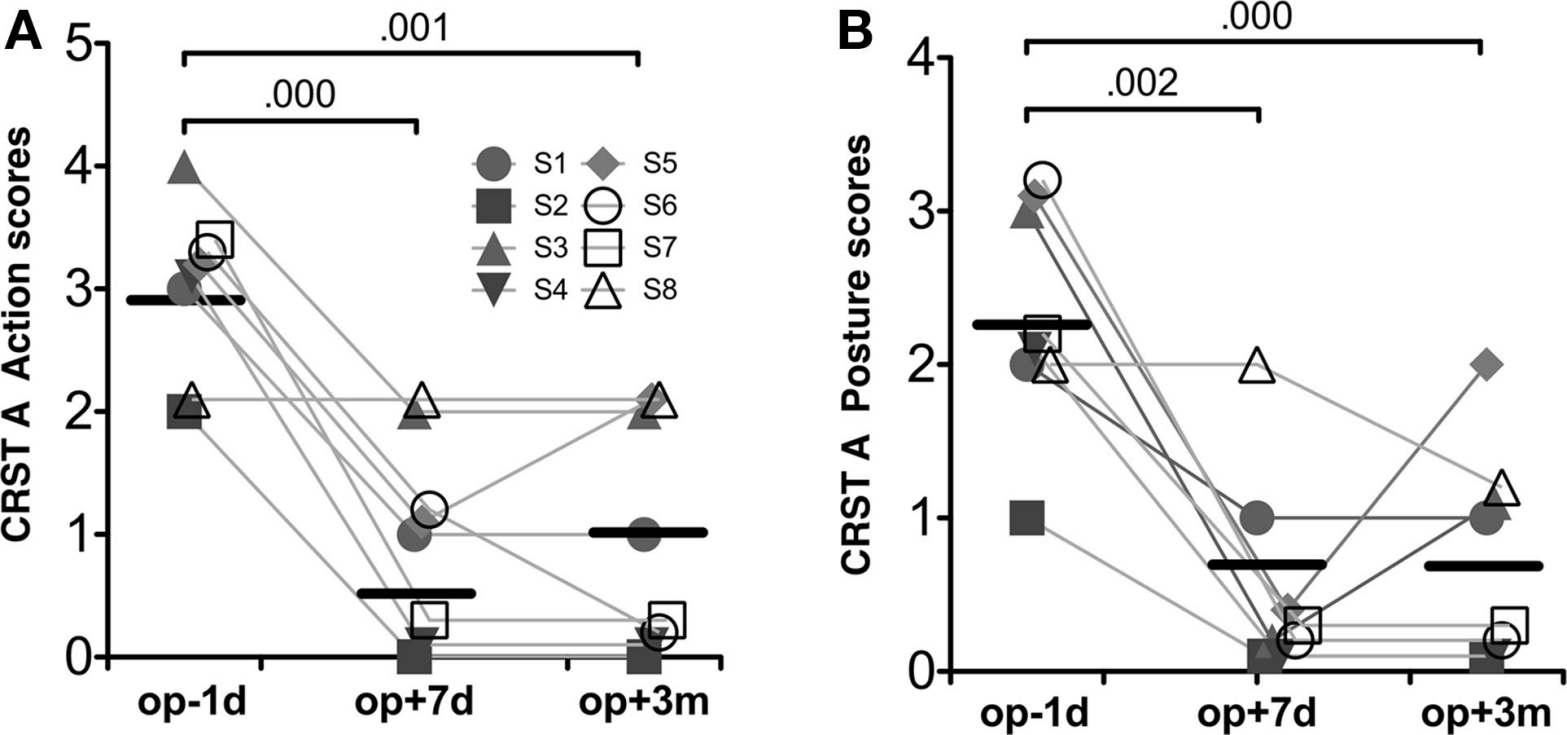
**Behavioral results**. CRST A action scores **(A)** and CRST A posture scores **(B)**. Repeated-measures ANOVA for both scores were significant session effects (*p* < 0.001). The numbers over the line indicate *p*-values for *post hoc* analysis.

Table 1 summarizes the demographic data for the patients in this study. The repeated-measures ANOVA for CRST A action scores and posture scores showed significant improvement after MRgFUS treatment [*F*(1.63, 11.40) = 28.00, *p* = 0.000, and *F*(2, 14) = 19.08, *p* = 0.000, respectively] (Figure 3).

**Table 1:** **Demographic data (CRST A scores for right hand)**.

ID	Sex	Age	Pre 1 day		Post 7 days		Post 3 months	
			Postural	Action	Postural	Action	Postural	Action
1	M	78	2	2	2	2	1	2
2	M	63	1	3	0	0	0	0
3	M	69	3	3	0	1	2	2
4	M	61	2	3	1	1	1	1
5	M	61	2	3	0	0	0	0
6	M	67	3	4	0	2	1	2
7	M	68	1	2	0	0	0	0
8	F	63	3	3	0	1	0	0

### Changes in the Motor-Tremor Network

**Figure 4:**
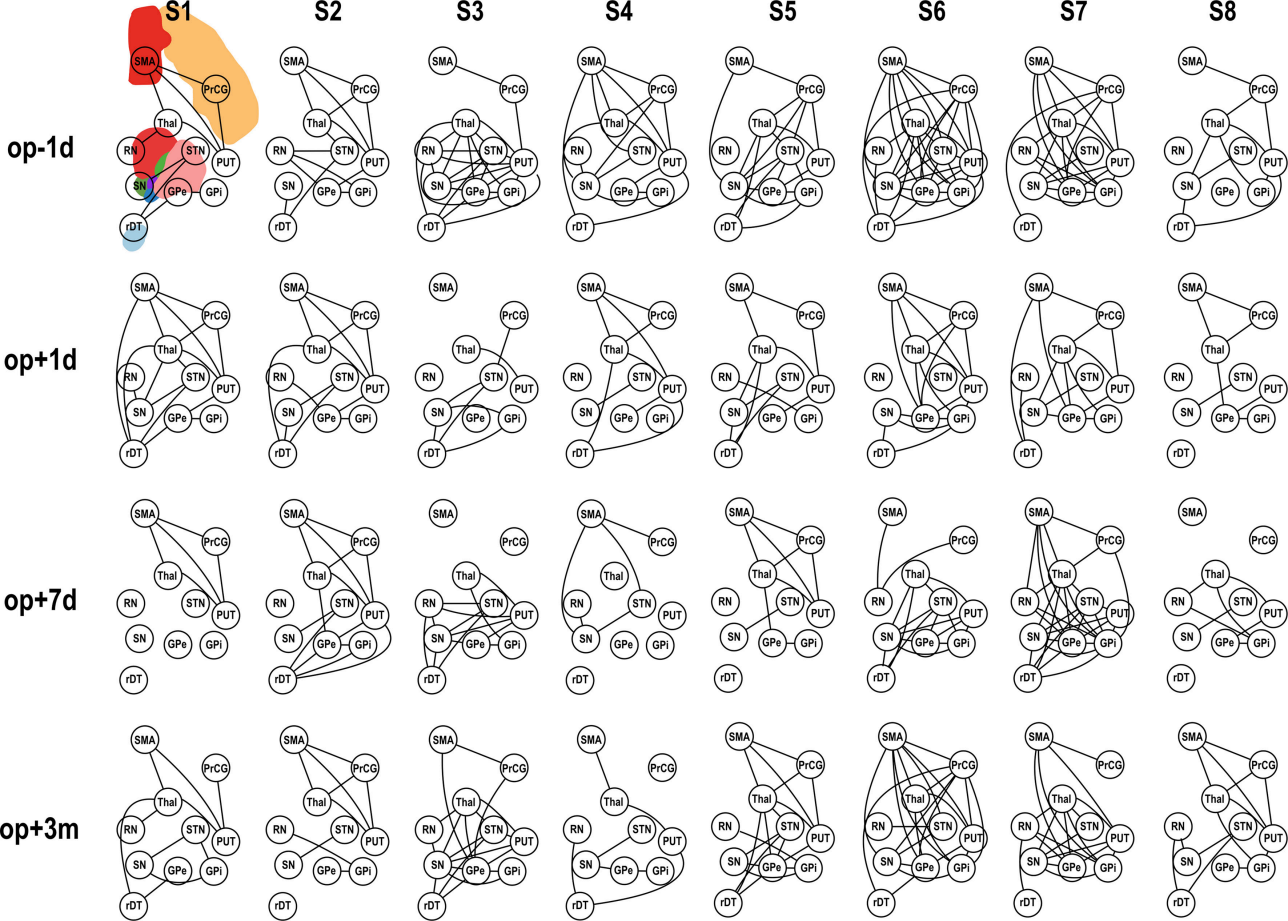
**Functional connectivity (FC) of motor subnetwork according to session**. op − 1d, op + 1d, op + 7d, and op + 3m indicate 1 day before operation, 1 day after operation, 7 days after operation, and 3 months after operation, respectively. Edges that passed the criteria of FDR *q* < 0.05 are displayed. PrcG, precentral gyrus; SMA, supplementary motor area; Thal, thalamus; Put, putamen; RN, red nucleus; GPe, external globus pallidum; GPi, internal globus pallidum; STN, subthalamic nucleus; SN, substantia nigra, in the left hemisphere; and rDT, right dentate nucleus in the cerebellum.

Application of pFC to the motor-tremor network showed few direct connectivity (pFC > 0) among nodes, indicating strong indirect or polysynaptic interactions in this network. Therefore, we evaluated graph-theoretic measures only for FC-based motor-tremor networks in the patients. Figure 4 displays changes in connectivity across sessions in the motor-tremor network of each patient at rest. Among heterogeneous functional connectivity across patients, patients had globally reduced connectivity within the motor-tremor network after thalamotomy.

**Figure 5:**
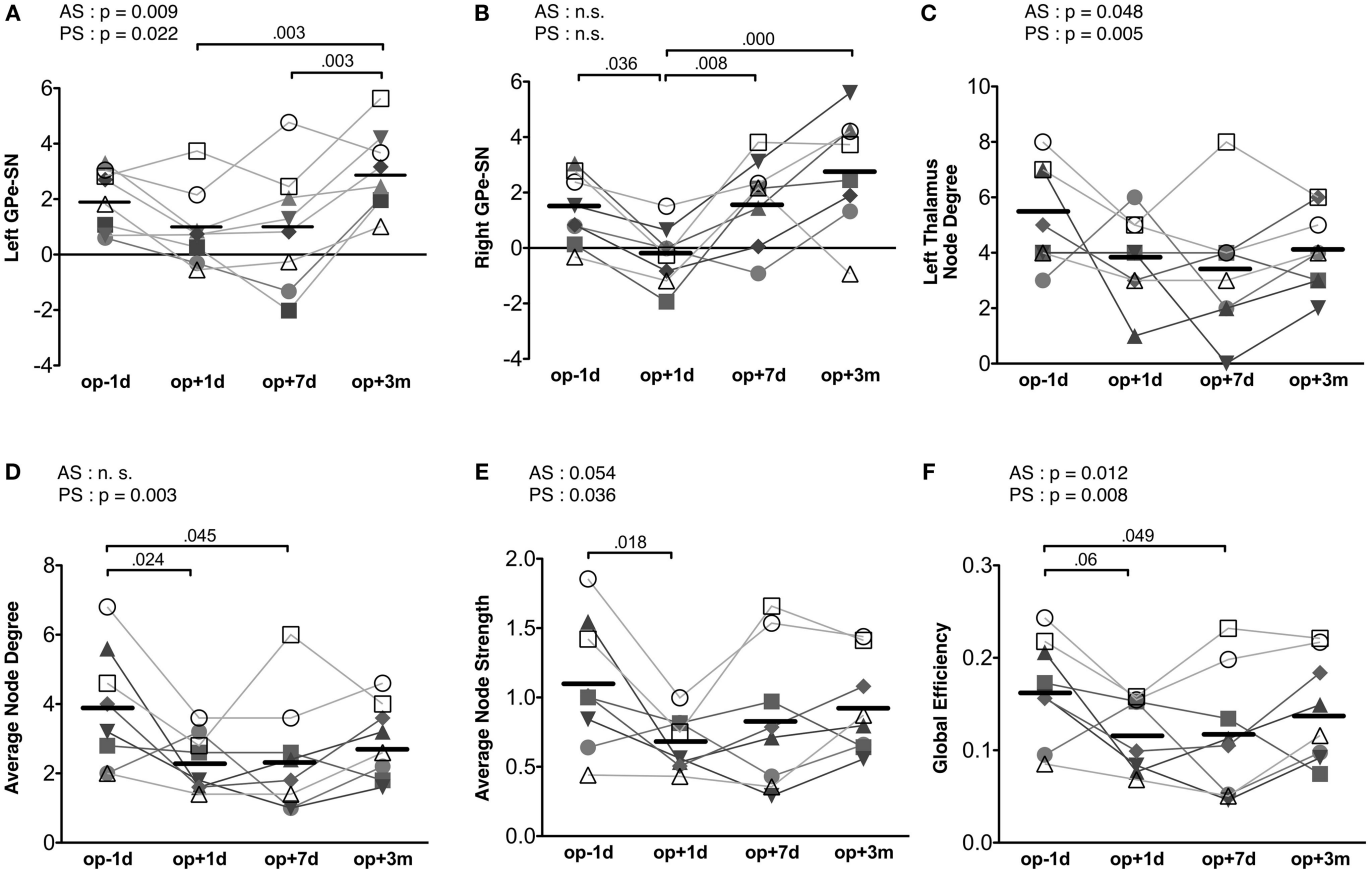
**Results of motor-tremor subnetwork analysis**. **(A)** Connectivity strengths (transformed to *Z*) between the left substantia nigra (SN) and left external globus pallidum (GPe). **(B)** Connectivity strengths between the right substantia nigra and right external globus pallidum. **(C)** Node degree in the left thalamus. **(D)** Average node degree in the motor-tremor network had significant session effect (*p* = 0.021) and CRST A posture score effect (*p* = 0.003). **(E)** The average node strength had a significant treatment effect (*p* = 0.027), a significant posture score effect (*p* = 0.036) and a tendency of action score effect (*p* = 0.054). **(F)** Global efficiency in the left motor-tremor network had a session effect (*p* = 0.033), CRST A posture score effect (*p* = 0.008), and CRST A action score effect (*p* = 0.0124). The numbers over the line indicate *p*-values for *post hoc* analysis. AS, CRST A action score; PS, CRST A posture score.

Repeated-measures ANOVA shows that the connectivity strength connecting the left substantia nigra and left external globus pallidum had a significant treatment effect [*F*(3, 21) = 7.73, *p* = 0.001] (Figure 5A). The connectivity strength decreased immediately following surgery and increased 3 months later. The mixed effect model analysis of this connectivity strength shows a significant main effect of action scores [*F*(1, 14.70) = 9.00, *p* = 0.009], an interaction effect on time × action scores [*F*(1, 19.10) = 6.28, *p* = 0.021], a main effect of posture scores [*F*(1, 17.01) = 6.38, *p* = 0.022], and an interaction effect on time × posture scores [*F*(1, 19.44) = 4.98, *p* = 0.038]. The contralateral edge strength connecting the right substantia nigra and right external globus pallidum also shows significant changes [*F*(3, 21) = 10.30, *p* = 0.000] (Figure 5B). The strength of this edge decreased immediately after surgery and increased 3 months later compared with the baseline pre-surgical state. No significant effect of action and posture scores on the connectivity between the right substantia nigra and right external globus pallidum was detected in the mixed effect analysis.

When we analyzed node degree of the target region, i.e., the left thalamus, the action and posture scores had significant effects on the change in node degree of the left thalamus [posture score: *F*(1, 12.40) = 11.67, *p* = 0.005; action score: *F*(1, 18.10) = 4.51, *p* = 0.048]; however, no significant main effect of session was observed in the repeated-measures ANOVA (Figure 5C).

The average node degree of the binarized motor-tremor network decreased immediately after MRgFUS and maintained a low level at 3 months after thalamotomy [*F*(3, 21) = 4.00, *p* = 0.021] (Figure 5D). A mixed effect model analysis showed a significant relationship between posture scores and the average node degrees in the patients [*F*(1, 17.38) = 12.24, *p* = 0.003]. The average node strength of the weighted motor-tremor network also decreased immediately after surgery and was maintained at a significantly lower level at 7 days after treatment [*F*(3, 21) = 3.72, *p* = 0.027] (Figure 5E). A mixed effect model analysis showed a significant action score effect [*F*(1, 15.20) = 4.34, *p* = 0.054] and posture score effect [*F*(1, 13.98) = 5.39, *p* = 0.036] on the average node strength. The global efficiency of the motor networks also had a significant treatment effect [*F*(3, 21) = 3.52, *p* = 0.033] (Figure 5F). Global efficiency decreased immediately after surgery and was maintained at lower level by 7 days after surgery. The action and posture scores had significant effects on global efficiency [posture scores: *F*(1, 13.95) = 9.35, *p* = 0.008, action scores: *F*(1, 15.39) = 8.01, *p* = 0.013].

### Changes in the Whole Brain Network

**Figure 6:**
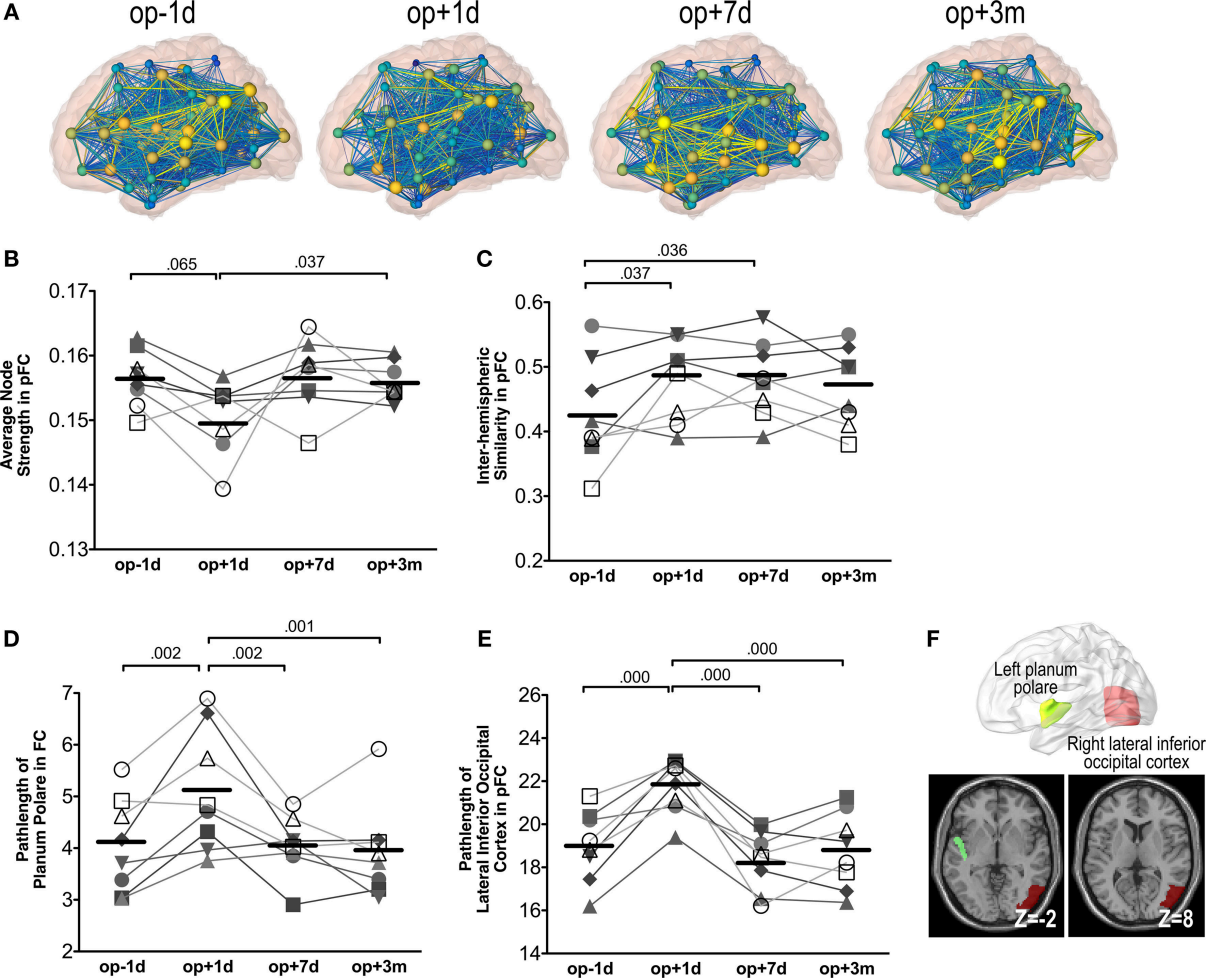
**Whole brain network analysis results**. **(A)** An exemplary display of network changes in the whole brain functional network of a patient (S6). **(B)** The average node strength over positive pFC network. **(C)** The inter-hemispheric similarity in partial-correlation functional connectivity matrix (pFC). **(D)** The path-length at the planum polare over FC. **(E)** The path-length at the lateral inferior occipital cortex over pFC. The numbers over the line indicate *p*-values for *post hoc* analysis. **(F)** Locations of the planum polare and the lateral inferior occipital cortex.

Figure 6A shows an example of network changes after thalamotomy in a patient. Repeated-measures ANOVA of the average node strength of pFC networks reveals significant changes after treatment [*F*(3, 21) = 3.65, *p* = 0.029], showing a tendency of reduction right after thalamotomy (*p* = 0.065, Tukey’s HSD) but increase to the pre-operative level by 7 days later. Action and posture scores had no significant effects on the average node strength (Figure 6B).

Repeated-measures ANOVA also shows significant changes in the inter-hemispheric similarity between the left and right hemisphere subnetworks of pFC [*F*(3, 21) = 3.87, *p* = 0.024]. The inter-hemispheric similarity increased immediately after thalamotomy (*p* = 0.037, Tukey’s HSD) and maintained by 3 months after treatment. However, there were no significant effects of action and posture scores on the inter-hemispheric similarity (Figure 6C).

We found significant changes in the path-length of FC in the left planum polare [*F*(3, 21) = 9.30, *p* = 0.000] (Figure 6D) and in the path-length of pFC in the right lateral inferior occipital cortex [*F*(3, 21) = 18.27, *p* = 0.000] (Figure 6E). The path-length in the left planum polare and the right lateral inferior occipital cortex (Figure 6F) increased immediately after thalamotomy but returned to the pre-operative level by 7 days after surgery. Action and posture scores had no significant effects on the path-lengths of the left planum polare and the right lateral inferior occipital cortex. All statistical analysis are summarized in Table 2.

**Table 2:** **Statistical result of network properties**.

Target		Longitudinal changes						Correlation with CRST A scores (mixed effect model analysis)	
		Repeated-measures ANOVA	op − 1d	op + 1d	op + 7d	op + 3m	*Post hoc* (Tukey’s HSD)	Action	Posture
Behavior	CRST A score (action)	*F*(1.6,11.4) = 28.00, *p* = 0.000	2.87 (0.64)	–	0.87 (0.83)	0.87 (0.99)	op − 1d > op + 7d: *p* < 0.001	–	–
							op − 1d > op + 3m: *p* = 0.001		
	CRST A score (posture)	*F*(2,14) = 19.08, *p* = 0.000	2.25 (0.71)	–	0.37 (0.74)	0.63 (0.74)	op − 1d > op + 7d: *p* = 0.002	–	–
							op − 1d > op + 3m: *p* < 0.001		

Motor-tremor network	Left GPe-SN	*F*(3,21) = 7.73, *p* = 0.001	2.01 (1.11)	0.95 (1.40)	0.97 (2.19)	3.02 (1.47)	op + 1d < op + 3m: *p* = 0.003	*F*(1,14.70) = 9.00, *p* = 0.009	*F*(1,17.01) = 6.38, *p* = 0.022
							op + 7d < op + 3m: *p* = 0.003		
	Right GPe-SN	*F*(3,21) = 10.30, *p* = 0.000	1.39 (1.24)	−0.25 (1.07)	1.76 (1.55)	2.81 (2.06)	op − 1d > op + 1d: *p* = 0.036	N.S.	N.S.
							op + 1d < op + 3m: *p* = 0.000		
							op + 1d < op + 7d: *p* = 0.008		
	
	Average node degree	*F*(3,21) = 4.00, *p* = 0.021	3.88 (1.72)	2.33 (0.83)	2.48 (1.68)	2.95 (1.08)	op − 1d > op + 1d: *p* = 0.024	N.S.	*F*(1,17.38) = 12.24, *p* = 0.003
							op − 1d > op + 7d: *p* = 0.045		
	Average node strength	*F*(3,21) = 3.72, *p* = 0.027	1.09 (0.48)	0.68 (0.20)	0.84 (0.52)	0.93 (0.34)	op − 1d > op + 1d: *p* = 0.018	*F*(1,15.20) = 4.34, *p* = 0.054	*F*(1,13.98) = 5.39, *p* = 0.036
	Global efficiency	*F*(3,21) = 3.52, *p* = 0.033	0.17 (0.05)	0.12 (0.04)	0.12 (0.07)	0.14 (0.06)	op − 1d > op + 1d: *p* = 0.060	*F*(1,15.39) = 8.01, *p* = 0.0124	*F*(1,13.95) = 9.35, *p* = 0.008
							op − 1d > op + 7d: *p* = 0.049		
	
	Node degree of left thalamus	N.S.	5.25 (1.83)	3.88 (1.55)	3.38 (2.33)	4.13 (1.46)	–	*F*(1,18.10) = 4.51, *p* = 0.048	*F*(1,12.40) = 11.67, *p* = 0.005

Whole brain network	Inter-hemispheric similarity of pFC	*F*(3,21) = 3.87, *p* = 0.024	0.43 (0.081)	0.48 (0.062)	0.48 (0.059)	0.47 (0.060)	op − 1d < op + 1d: *p* = 0.037	N.S.	N.S.
							op − 1d < op + 7d: *p* = 0.036		
	Average strength of pFC	*F*(3,21) = 3.65, *p* = 0.029	0.1565 (0.0044)	0.1506 (0.0056)	0.1571 (0.0055)	0.1559 (0.0030)	op − 1d > op + 1d: *p* = 0.065	N.S.	N.S.
							op + 1d < op + 3m: *p* = 0.037		
	Path-length of left planum polare in FC	*F*(3,21) = 9.30, *p* = 0.000	4.05 (0.92)	5.10 (1.19)	4.05 (0.57)	3.93 (0.90)	op − 1d < op + 1d: *p* = 0.002	N.S.	N.S.
							op + 1d > op + 7d: *p* = 0.002		
							op + 1d > op + 3m: *p* = 0.001		
	Path-length of right lateral inferior occipital cortex in pFC	*F*(3,21) = 18.27, *p* = 0.000	19.01 (1.65)	21.80 (1.26)	18.30 (1.36)	18.78 (1.77)	op − 1d < op + 1d: *p* = 0.000	N.S.	N.S.
							op + 1d > op + 7d: *p* = 0.000		
							Op + 1d > Op + 3m: *p* = 0.000		

*GPe, external globus pallidum; SN, substantia nigra; N.S., not significant; FC, functional connectivity-based on Pearson correlation; pFC, partial-correlation functional connectivity. Values in the op − 1d, op + 1d, op + 7d, and op + 3m indicate mean and SDs*.

## Discussion

Using a minimally invasive MRgFUS technique and resting state connectivity analysis, we investigated longitudinal alterations of functional networks after a deliberate focal lesion in the treatment of essential tremor. We had two main findings. First, MRgFUS thalamotomy leads to global changes in both the motor network and whole brain network, beyond the single target region. Second, the networks show differential longitudinal time courses, transient changes followed by immediate recovery and long-lasting changes. Symptom-related changes are mainly found in the circuit of the motor network while transient changes are mainly observed in the whole brain network.

### Symptom-Related Changes in the Motor-Tremor Network at Rest

Previous studies on the neural mechanism of essential tremor revealed the importance of motor circuitry, including the thalamus (40, 41), motor cortex (42), cerebellum (43, 44), and red nucleus (45). An fMRI study showed the involvement of the motor area, thalamus, red nucleus, and globus pallidum (46), implying altered cerebello-thalamo-cortical signaling (47, 48).

Thalamotomy, in the treatment of essential tremor, is thought to suppress neural involvement of the Vim, similar to antiepileptic medications, such as primidone (49). As a node participating in this circuit, ablation of the Vim of the thalamus was expected to have an effect at the circuitry level of the motor network, which was observed in this study. This study shows that reduced thalamic activity not only changes direct thalamo-cortical connection but also modulates indirect connections in the closed loop of the motor network. Indeed, we found a tendency toward symptom (posture score)-related changes in the node degree of the left thalamus in the mixed effect analysis, indicating a reduced number of connections with other areas in the motor network. However, the treatment effect was not confined to connectivity in the target lesion, i.e., the left thalamus.

The average node degree in the motor-tremor network decreased immediately after treatment, a level that was maintained by 3 months after the treatment. A similar trend was seen in the average node strength. The reduced average nodal degree and strength of FC in the motor network implies reduced interactions between nodes via direct or indirect polysynaptic connections in the motor network. This is also reflected in the reduced global efficiency (reduced hyper-interactions) of the network. This result is consistent with a diffusion tensor imaging study that reported a continuous decrease in the fractional anisotropy in the lesion and distant areas, including the sensory motor area, corticospinal tract, posterior arm of the internal capsule, and cerebellum, reflecting the degeneration of fibers (50). We also found significant CRST A posture score effects on the average node degree and global efficiency, i.e., decreased posture score and reduced node degree within the motor network. This result suggests that thalamotomy regulates interaction among areas in the motor network, which alleviates the tremor symptoms.

Among the reduced interactions within the motor network, the left substantia nigra-external globus pallidum connectivity had a significant treatment effect. The substantia nigra, located in the mesencephalon (midbrain), and the external part of the globus pallidum, the major component of the basal ganglia, are known to be indirectly connected via striatal medium spiny cells (51). How does a thalamotomy induce changes in the connectivity between these two regions, which are known to have connectivity toward the thalamus, not directly from the thalamus? It should be noted that functional connectivity can exist even in the absence of direct anatomical projections, possibly via polysynaptic connections. A closed extra-pyramidal motor loop, i.e., thalamus–cortex–subthalamic nucleus–substantia nigra–striatum–external globus pallidum may be a potential candidate for this polysynaptic effect. In line with the fact that tremor in the Parkinson’s disease can be controlled by both Vim and subthalamic nucleus deep brain stimulation (52), this study suggests a role for Vim thalamotomy in the regulation of the extra-pyramidal circuit.

The reduced edge strengths between the ipsilateral substantia nigra and external globus pallidum returned to and remained at the baseline state by 7 days after treatment. By contrast, the connectivity changes between the right substantia nigra and external globus pallidum contralateral to the lesion followed different time courses and demonstrated a transiently reduced but a subsequently increasing pattern, implying on-going compensatory reorganization.

In the motor network, we could not identify consistent changes in a particular edge across patients except for the substantia nigra-external globus pallidum connectivity. Individuals may utilize different strengths of edges among nodes, before and after treatment, during the resting state. Instead, the treatment modulation was reflected in the sum of all interactions, i.e., average node degrees across the patients.

### Transient Alterations in the Global Brain Network

Focal lesioning of the Vim of the thalamus causes remote and global changes in the brain network. The Vim of the thalamus is generally known as a way point from the cerebellum to the primary motor cortex. The probabilistic fiber tractography of DTI has shown interconnection between the Vim and both the primary motor cortex and the contralateral cerebellum (53, 54). Despite these known connections in the motor pathways, recent network analyses have shown that thalamus is one of important hubs in the whole brain (55, 56). Thus, we can easily consider that thalamotomy affects not only motor system but also the global brain system.

We found that the effects of thalamotomy on the global brain network were mostly transient. The ablation of the Vim of the left thalamus decreased the whole brain average node strength of pFC immediately after surgery, which then returned to baseline by 7 days after the treatment, indicating transient suppression of direct interactions over the brain.

In the node level property, we observed transient changes in the path-length (over the whole brain FC) of the left planum polare, a portion of the superior temporal gyrus near the temporal pole in the Harvard-Oxford atlas. The right lateral inferior occipital cortex also showed an increased path-length of pFC. The left planum polare is known to be involved in language and music performance or singing (57–60) and in movement-related disorders (61, 62), implicating an association with motor skill. The lateral inferior occipital cortex is known to be involved in goal-directed movements of the observer’s body parts (63) and is influenced by motor region during mental imagery (64). Since the path-length of a node represents the average number of edges to travel to any node in the whole brain network from the given node, increased path-lengths of the left planum polare and the right lateral inferior occipital cortex imply their losses of “short-cuts” (strong interactions) or reduced functional interactions on the way to other brain regions.

We also found immediate reduction of the inter-hemispheric network asymmetry in the whole brain pFC network, which maintains reduced asymmetry (or increased symmetry) afterwards. Considering the fact that essential tremor generally occurs unilaterally (65), reduced Vim activity in the left brain may lessen ipsilateral hyper-interactions to the level seen in the contralateral hemisphere.

All these results on the whole brain network correspond to transient connectional and connectomal diaschisis effects due to focal lesion (66) at the left thalamus, indicating “selective change in coupling between two nodes of a defined network, involving areas distant from the lesion” (connectional diaschisis) and “changes in the structural functional connectome, including disconnections between and reorganization of subgraphs, involving areas distant from the lesion” (66). This transient diaschisis may be a result of inhibitory or excitatory effects of a brain lesion (66).

We cannot disregard the possibility of lesioning not only the motor-related area in the thalamus but also other nuclei that relay to other brain regions. Transient global networks may also be attributable to local brain edema or temporary neuronal knock-out dysfunctions, which are unintended functional changes due to lesioning. In any case, transient changes in the global network other than the motor area could be an evidence for recovery of unintended thalamotomy effects.

### Direct Thalamic Connectivity and Functional Connectivity Measures

Although we found generally reduced connectivity (node degree) at the thalamus in the motor-tremor network, we did not find a significant change in the direct connectivity between the thalamus and the motor cortical area. This may be explained by insensitivity of the connectivity measure using rs-fMRI. Functional connectivity, defined by cross-correlation between the time series of two nodes, cannot differentiate reduced activity from the baseline as long as two BOLD time series are time synchronized. Since the lesion in the Vim reduces but not completely removes the regional activity in this area, the synchronous BOLD signals in the thalamus and motor cortex may be maintained. To investigate this type of change in detail, we may need to analyze effective connectivity, for example, through dynamic causal modeling (67). We cannot also ignore heterogeneous responses to thalamotomy across patients, which reduce statistical power at each node or edge. Since a function can be implemented by different configurations of circuits, the effect of the thalamotomy on the motor circuit may not be generalized in part but be reflected in the multivariate index, such as average node degree.

As a measure of functional connectivity, we used partial correlation (pFC) as well as Pearson correlation coefficient (FC). The Pearson correlation coefficient is a conventional way of defining a functional edge between two distinct regions and reflects both direct interactions and indirect interactions from polysynaptic induction, common modulatory effects, or common feed-forward projections via the thalamus (68). Instead, partial correlation is more efficient in revealing direct associations between the brain areas. In the whole brain network, the lesion at the Vim affects the direct associations (pFC) more than both direct and indirect associations (FC), as shown in the results of the average node strength and inter-hemispheric similarity.

### Limitations

This study has several limitations. A small number of patients with extreme tremor may lead to low statistical power considering the heterogeneity of the subjects. Furthermore, this study is based on the resting state network, which may not be directly compatible with motor performance. This explains the result that the node degree of the left thalamus, average degree, and global efficiency had stronger posture score effects than action score effects (Figure 5). However, the longitudinal network changes during motor performance are still open to questions. Since we acquired data at 1 day, 7 days, and 3 months after treatment, the precise timing of the network changes particularly between 1 day and 3 days following surgery could not be differentiated. This study also lacks data from longitudinal changes in the healthy control groups. However, recent studies have shown the reproducibility of the functional networks defined by rs-fMRI in multiple sessions (69), days (70), and 1 year apart (71). This study was also limited in the identification of the subcortical motor area, based on the atlas used, which may have led to contamination from neighboring nuclei, for example, the red nucleus and subthalamic nucleus and, thus, cause construction errors for the functional network. This study reports the longitudinal changes that occurred after a time period of up to 3 months. Further long-term longitudinal studies in these subjects would be useful to see the pattern of evolution of the changes in the networks.

### Summary

In summary, we demonstrated that a focal lesion in the Vim of the thalamus induced by using minimally invasive MRgFUS could lead to changes in both the global brain network and motor circuit, and does not simply alter activity or connectivity changes in the target region. Using longitudinal data, we could identify two different types of network alterations in the time course: immediate changes followed by short-term symptom-unrelated recovery and long-lasting symptom-related alterations. This study shows the usefulness of the combination of MRgFUS and connectivity analysis using rs-fMRI in exploring connectional and connectomal diaschisis identified following thalamotomy in the treatment of essential tremor.

## Author Contributions

Conceived and designed the experiments: JWC and HJP. Performed the experiments: CJ and WSC. Analyzed the data: CJ and CP. Contributed reagents/materials/analysis tools: CP. Wrote the paper: CJ and HJP. Revised : JWC.

## Conflict of Interest Statement

The authors declare that the research was conducted in the absence of any commercial or financial relationships that could be construed as a potential conflict of interest.

## Footnotes

^1^http://neuroimage.yonsei.ac.kr/mnet.

^2^http://www.fmrib.ox.ac.uk/fsl.

^3^http://www.fil.ion.ucl.ac.uk/spm/.

^4^http://people.bss.phy.cam.ac.uk/~rea1/AIRWC.html.
